# From Yeast Probiotics to Postbiotics: A Continuum Model and Yeast-Specific Functional Perspective

**DOI:** 10.3390/cells15151320

**Published:** 2026-07-23

**Authors:** Gabriela Żywczak, Pattanan Songdech, Roksolana Vasylyshyn, Justyna Ruchała

**Affiliations:** 1 Faculty of Biotechnology, Medical College, University of Rzeszów, 35-601 Rzeszów, Poland; gz122267@stud.ur.edu.pl (G.Ż.); vasylyshyn.r.v@gmail.com (R.V.); 2 Excellent Research Laboratory for Yeast Innovation, Division of Biochemical Technology, School of Bioresources and Technology, King Mongkut’s University of Technology Thonburi (KMUTT), Bangkok 10150, Thailand; pattanan.s@psu.ac.th; 3 Department of Molecular Genetics and Biotechnology, Institute of Cell Biology, National Academy of Sciences of Ukraine, 79005 Lviv, Ukraine

**Keywords:** yeast-biotic continuum, *Saccharomyces cerevisiae* var. *boulardii*, mycobiome, next-generation biotics, extracellular vesicles, immune modulation, gut barrier function

## Abstract

Growing interest in probiotics and postbiotics has expanded research beyond living microorganisms and highlighted the functional importance of microbial cells, metabolites, and structural components. However, current conceptual frameworks still primarily focus on bacteria, while yeasts, despite their industrial, nutritional, and clinical importance, remain underrepresented. Owing to their eukaryotic organization, complex cell wall architecture, specialized secretory pathways, and ability to produce diverse bioactive molecules, yeasts exhibit functional properties that are distinct from those described for bacterial probiotics and postbiotics. In this review, we examine the relationship between yeast probiotics and yeast-derived postbiotics and propose the concept of a yeast-biotic continuum, which describes biological functionality as a dynamic progression from living cells, through active metabolism and released bioactive factors, to inactivated cells and postbiotic preparations. In this approach, postbiotics are viewed not as static end products but as steps in a continuous biological process influenced by cell viability, environmental conditions, and interactions with the surrounding microbiota. We discuss major yeast-derived bioactive components, including β-glucans, mannoproteins, metabolites, peptides, and extracellular vesicles, together with their roles in immune modulation, epithelial barrier support, pathogen interference, and fungi-bacteria-host interactions. Finally, we highlight current challenges, emerging technologies, and future opportunities for yeast-derived biotics.

## 1. Introduction

The growing recognition of the human microbiome as a key determinant of health has intensified interest in microbiome-targeted interventions, including probiotics, prebiotics, synbiotics, and, more recently, postbiotics and next-generation biotics [1,2]. While early studies focused primarily on the administration of live microorganisms, a growing body of evidence suggests that health-promoting effects may also be indirectly mediated by non-viable microbial cells, structural cell components, and microbially derived metabolites. This paradigm shift has led to the emergence of postbiotics, one of the fastest-growing areas in microbiome science and functional food research [3,4]. This growing interest in postbiotics stems from a number of practical and scientific considerations. Unlike conventional probiotics, postbiotic preparations may offer improved stability during processing and storage, greater safety in susceptible populations, and more consistent functional properties due to the absence of viability-related variability [4]. Furthermore, advances in microbiome research have revealed that many biological effects traditionally attributed to live microorganisms may actually result from the metabolites they secrete, as well as extracellular structures or microbial-associated molecular patterns that remain biologically active even after cellular inactivation [3,4]. Consequently, contemporary research is increasingly focused on understanding microbial functionality beyond mere viability.

Despite the rapid development of this field, current concepts of probiotics, postbiotics, and next-generation biotics are still largely shaped by bacterial models. Most studies exploring mechanisms, as well as regulatory definitions and conceptual frameworks, have been developed using lactic acid bacteria, bifidobacteria, and other bacterial taxa as reference organisms [1,2]. Consequently, the biodiversity of nonbacterial microorganisms remains underrepresented in both discussions of mechanisms of action and concepts for microbiome-based interventions.

**Figure 1:**
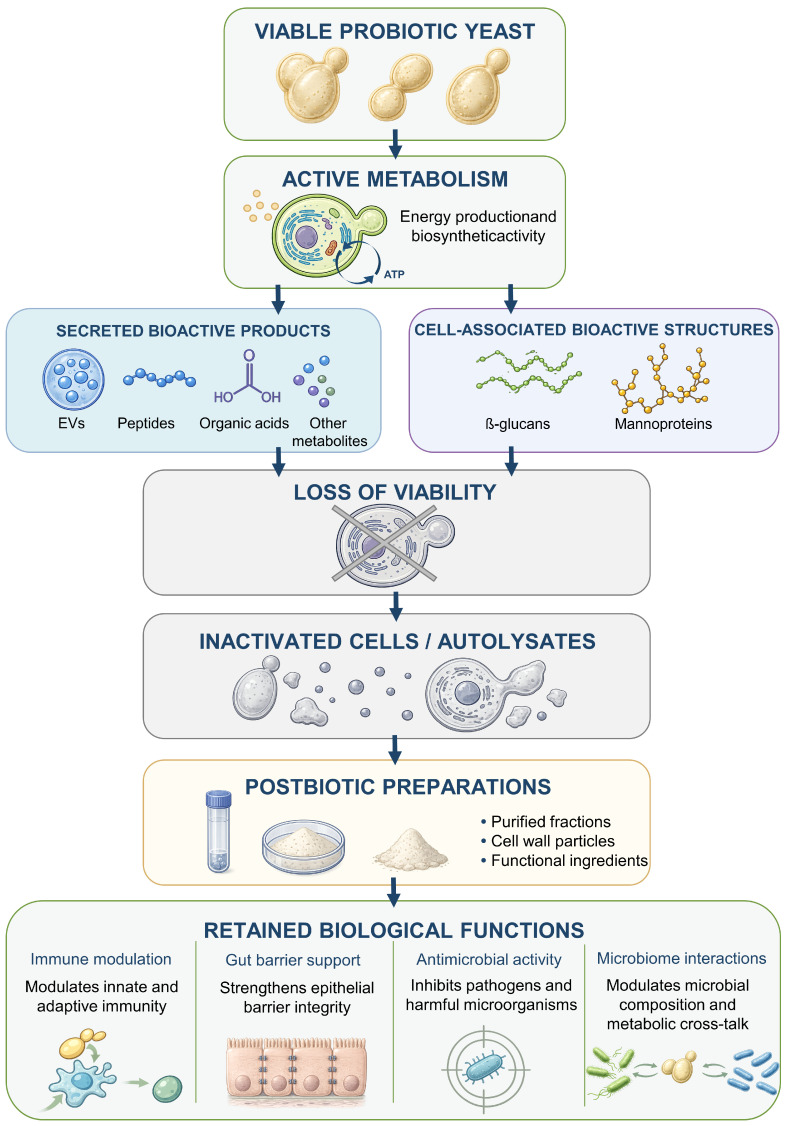
The yeast-biotic continuum: from viable yeast cells to postbiotic functionality. Conceptual representation of the yeast-biotic continuum proposed in this review. The model illustrates the progressive transition from viable probiotic yeast cells through active metabolism and the production of secreted bioactive factors, including extracellular vesicles (EVs), peptides, organic acids, and other metabolites, to non-viable cells, autolysates, and postbiotic preparations. In parallel, cell wall-associated structures, particularly β-glucans and mannoproteins, remain biologically active following loss of viability and contribute to the functional properties of yeast-derived postbiotics.

Among these overlooked microbial groups, yeasts are particularly important. Yeasts have a long history of safe use in biotechnology and fermentation, hold Qualified Presumption of Safety (QPS) status issued by EFSA for several species, and are among the most thoroughly studied nonbacterial probiotics [5,6]. In particular, *Saccharomyces cerevisiae* var. *boulardii* has demonstrated clinical efficacy in the prevention and treatment of antibiotic-associated diarrhea, recurrent *Clostridioides difficile* infections, and other gastrointestinal disorders, making this species one of the best-characterized probiotic microorganisms currently available [7,8]. In addition to *S. cerevisiae* var. *boulardii*, an increasing number of yeast species, including representatives of *Kluyveromyces*, *Debaryomyces*, *Meyerozyma*, and other genera, are being investigated for their probiotic and biotherapeutic potential [9,10,11]. Importantly, yeasts differ fundamentally from bacteria in their eukaryotic cellular organization, cell wall composition, secretory pathways, and ecological interactions. These differences influence how yeasts communicate with host tissues, interact with resident microbiota, and generate biologically active molecules [8,12]. However, most existing postbiotic frameworks still interpret yeast-derived functionality through concepts originally developed for bacterial systems, potentially omitting mechanisms unique to these particular fungal microorganisms (Figure 1).

In this review, we propose a yeast-centered perspective on probiotic and postbiotic functionality. Existing classifications generally distinguish microbial products according to viability status, separating live probiotics from inactivated cells and postbiotic preparations. However, many biologically relevant yeast-derived activities arise through a continuum of interconnected processes that begin with active cellular metabolism and extend beyond cellular viability through the persistence of secreted molecules, extracellular vesicles, and structurally conserved cell wall components. To address this conceptual gap, we propose the yeast-biotic continuum, a framework that views probiotic and postbiotic functionality as successive and overlapping functional states rather than discrete categories. Using this perspective, we discuss the molecular mechanisms underlying yeast-derived biological activity and examine how a yeast-centered framework may complement and extend existing probiotic and postbiotic paradigms.

## 2. Yeasts as Probiotics: Beyond the Classical Model

### 2.1. Comparison of Bacterial and Yeast Probiotics

Bacterial probiotics, particularly representatives of the genera *Lactobacillus*, *Lacticaseibacillus*, *Bifidobacterium*, and related taxa, have long served as the principal models for probiotic research and clinical applications. To exert probiotic effects, bacterial strains must first survive exposure to gastric acid, bile salts, and digestive enzymes during gastrointestinal transit and transiently adhere to the intestinal mucosa, enabling interactions with the resident microbiota and host tissues [13,14]. These and other characteristics, including antimicrobial activity and safety, are routinely evaluated during the selection of new probiotic strains [15,16,17]. Once established, they promote host health through multiple mechanisms, including modulation of the gut microbiota and immune responses, production of vitamins and antimicrobial compounds, reinforcement of intestinal barrier function, competitive exclusion of pathogens, and prevention of gastrointestinal disorders such as antibiotic-associated diarrhea [18,19]. However, because probiotic bacteria coexist with other bacteria in the gastrointestinal tract, they may also participate in the horizontal transfer of antibiotic-resistance genes [20].

Yeasts offer a complementary probiotic strategy. As eukaryotic microorganisms, they lack the cellular targets of antibacterial antibiotics and are therefore naturally insensitive to these drugs. This property allows probiotic yeasts to be administered during antibiotic therapy without loss of viability and minimizes the risk of transferring antibiotic-resistance genes within the bacterial microbiota [21]. Several probiotic yeast strains, particularly *S. cerevisiae* spp. *boullardii* also tolerate acidic pH, bile salts, and other gastrointestinal stresses, allowing them to survive passage through the digestive tract [4].

**Figure 2:**
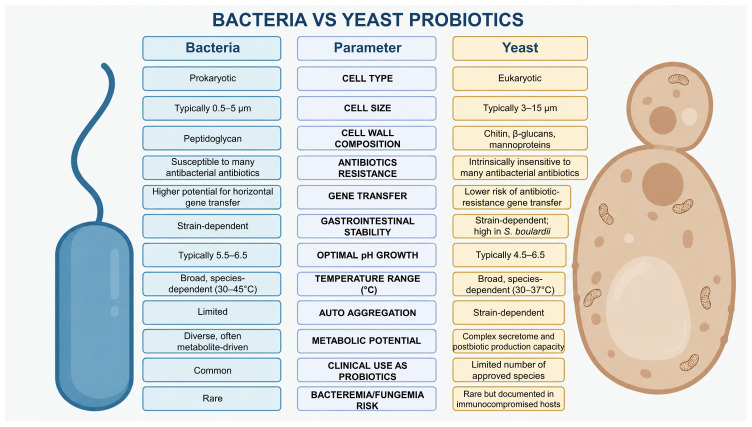
Schematic comparison of cellular architecture and biological characteristics of bacterial and yeast probiotics. Based on [4,12,20,21].

The unique properties of probiotic yeasts arise from their eukaryotic cellular organization (Figure 2). Unlike bacterial probiotics, yeast cells contain membrane-bound organelles, including the endoplasmic reticulum, Golgi apparatus, and mitochondria, together with a highly developed secretory system that enables complex post-translational protein modifications such as glycosylation [12]. In addition, the yeast cell wall differs fundamentally from the bacterial peptidoglycan envelope, being composed primarily of β-glucans, mannoproteins, and chitin. These structural and cellular features enable probiotic yeasts to produce extracellular glycoproteins, enzymes, extracellular vesicles, peptides, and other bioactive molecules that are absent or much less common in bacterial probiotics. Consequently, although bacterial and yeast probiotics share fundamental probiotic characteristics, probiotic yeasts communicate with the host and surrounding microbiota through several additional mechanisms that are specific to fungal cells.

### 2.2. Cellular Mechanisms of Action of Yeast Probiotics

**Figure 3:**
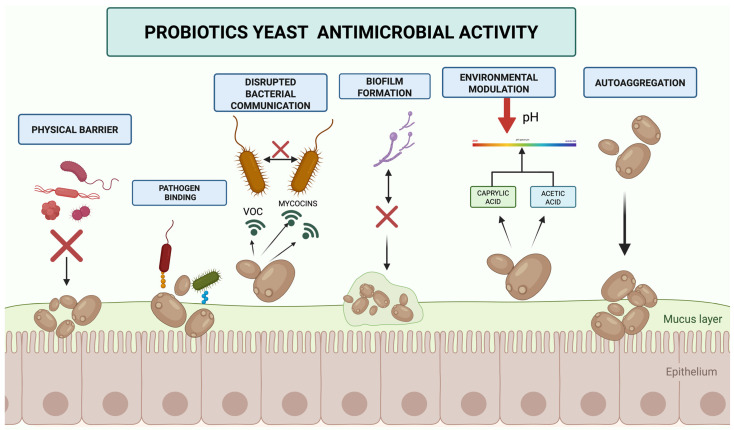
Antimicrobial activities and pathogen inhibition mechanisms mediated by probiotic yeasts. Adapted from [4,26,27,28,29] Alkalbani et al. (2022), Bahuguna et al. (2025), Buonanno et al. (2025), Franco (2024), and Scott et al. (2021).

The best-characterized probiotic yeast is *S. cerevisiae* var. *boulardii*, which has demonstrated clinical efficacy in the prevention and management of antibiotic-associated diarrhea, recurrent *C. difficile* infection, acute infectious diarrhea, and as an adjunct to *Helicobacter pylori* eradication therapy [7,12,22]. The beneficial effects of probiotic yeasts arise from multiple complementary mechanisms involving interactions with pathogens, the host immune system, the intestinal epithelium, and the resident microbiota (Figure 3). A major component of these interactions is the yeast cell wall, which is enriched in mannans and β-glucans. Yeast β-glucans are recognized by host pattern-recognition receptors, thereby activating innate immune responses and regulating macrophage and dendritic cell functions [4,23] (Franco, 2024; Makriyanni & Yanni, 2026). Cell wall mannans may additionally bind and sequester enteric pathogens, thereby reducing their adhesion to epithelial surfaces and facilitating their removal from the gastrointestinal tract [4,24]. The structure and biological activities of these cell wall components after cellular inactivation are discussed in Section 3.

Probiotic yeasts reinforce intestinal barrier function by preserving epithelial integrity, limiting pathogen translocation, and promoting epithelial regeneration following injury. These protective effects are associated with increased expression of tight-junction proteins, stimulation of mucus secretion by goblet cells, and maintenance of epithelial homeostasis. In *S. cerevisiae* var. *boulardii*, secreted enzymes further reduce pathogen-mediated damage by degrading bacterial toxins or modifying host receptors involved in toxin binding, particularly during infections caused by toxin-producing enteric pathogens such as *C. difficile* [7,8]. In addition, *S. cerevisiae* var. *boulardii* produces polyamines that promote enterocyte maturation, epithelial restitution, and brush-border enzyme activity, thereby supporting intestinal epithelial renewal [25].

Probiotic yeasts also exert direct antagonistic activity against intestinal pathogens through multiple complementary mechanisms. These include competitive exclusion for nutrients and adhesion sites, occupation of ecological niches, and secretion of extracellular bioactive compounds such as volatile organic compounds (VOCs), organic acids, antimicrobial proteins, and mycocins. Collectively, these metabolites inhibit pathogen growth, interfere with quorum sensing, and suppress biofilm formation [29]. Similar antagonistic mechanisms have been reported against fungal pathogens, particularly *Candida* spp., through inhibition of adhesion, biofilm development, and secretion of inhibitory metabolites [27,28]. Certain yeast-derived mycocins additionally exhibit antibacterial activity against clinically important Gram-positive and Gram-negative pathogens, highlighting the broad antimicrobial potential of probiotic yeasts [30]. Beyond direct antimicrobial effects, probiotic yeasts actively modulate host immune responses. Recognition of yeast cell wall components by immune cells stimulates cytokine production and contributes to balanced immune activation. This process is characterized by enhanced production of anti-inflammatory mediators, particularly interleukin-10 (IL-10), together with suppression of pro-inflammatory cytokines such as tumor necrosis factor-α (TNF-α) and interleukin-1β (IL-1β), thereby promoting immune homeostasis and reducing excessive intestinal inflammation [4,23].

Although probiotic yeasts colonize the gastrointestinal tract only transiently, they can influence the composition and metabolic activity of the resident microbiota by promoting beneficial microorganisms, competing with pathogens, and modifying the intestinal microenvironment through metabolite production [31]. These interactions may indirectly stimulate bacterial production of short-chain fatty acids (SCFAs), including acetate, propionate, and butyrate, which contribute to epithelial energy metabolism, maintenance of intestinal barrier integrity, and regulation of host immune responses [32].

Certain yeast strains additionally exhibit strong autoaggregation and coaggregation properties, facilitating persistence during gastrointestinal transit and promoting interactions with resident microbial communities [26]. Importantly, several probiotic functions are not strictly dependent on cellular viability. Structural cell wall components, secreted metabolites, extracellular proteins, and vesicle-associated factors may remain biologically active after cell inactivation. This functional continuity between viable cells and their bioactive derivatives provides the conceptual basis for the yeast-biotic continuum explored in the following sections.

### 2.3. Probiotic-Associated Traits in Saccharomyces and Non-Saccharomyces Yeasts

The expanding field of probiotic yeast research has demonstrated that probiotic potential extends far beyond *S. cerevisiae* var. *boulardii*, encompassing a growing diversity of food-associated and environmental yeasts with promising functional properties. Advances in strain isolation, molecular identification, and functional characterization have revealed numerous non-*Saccharomyces* species capable of surviving gastrointestinal conditions, interacting with the intestinal microbiota, producing bioactive metabolites, and modulating host physiology. Importantly, these functional attributes are highly strain-specific and therefore cannot be generalized at the species level. Consequently, the selection of candidate probiotic yeasts requires comprehensive phenotypic, genomic, functional, and safety evaluation prior to their application in food or clinical settings. Representative yeast strains exhibiting experimentally validated probiotic-associated traits are summarized in Table 1 [1,9,33].

Among probiotic yeasts, *S. cerevisiae* var. *boulardii* CNCM I-745 remains the reference strain owing to its extensive clinical validation and documented efficacy in the prevention and management of antibiotic-associated diarrhea, recurrent *C. difficile* infection, acute infectious diarrhea, and as an adjunct during *H. pylori* eradication therapy [7,22]. Nevertheless, experimental evidence accumulated over the last decade has demonstrated that comparable functional characteristics are distributed across numerous non-*Saccharomyces* yeasts. These observations indicate that probiotic functionality represents a broadly conserved physiological capability rather than a feature restricted to a single taxonomic lineage.

A fundamental requirement for probiotic activity is survival during gastrointestinal transit. Several food-derived non-*Saccharomyces* yeasts satisfy these criteria. *Wickerhamomyces anomalus* HN1, isolated from Egyptian cheese, remained viable in the presence of up to 2.0% bile salts, demonstrating exceptional resistance to intestinal stress conditions [34]. Similarly, *Torulaspora delbrueckii* strains isolated from spontaneously fermented foods survived simulated gastric juice while exhibiting pronounced antioxidant activity [9,35]. Comparable characteristics have also been reported for *Pichia kudriavzevii* strain 5S5, which combined excellent survival under simulated gastrointestinal conditions with high cell surface hydrophobicity and efficient adhesion to intestinal epithelial cells, comparable to those of *S. cerevisiae* var. *boulardii* CNCM I-745 [36].

The diversity of candidate probiotic yeasts has expanded considerably with the characterization of novel isolates from both food-associated and environmental sources. *Lachancea thermotolerans* Lt101, *Kazachstania unispora* Kum3-B3, *M. guilliermondii* Mg112, *M. caribbica* Mc58, and *D. hansenii* Dh36 remained viable throughout the fermentation of plant-based beverages, confirming their previously reported functional characteristics in distinct food matrices [9]. Similarly, environmental isolates of *L. thermotolerans* CMGB-ST12 tolerated both acidic (pH 3.0) and highly alkaline conditions while maintaining growth at 37 °C, indicating physiological robustness under environmental stress [37]. Together, these findings considerably broaden the spectrum of candidate probiotic yeasts beyond conventional dairy-associated species and demonstrate that functionally relevant strains can be recovered from diverse ecological niches.

Beyond gastrointestinal survival, effective probiotic candidates should demonstrate the ability to interact with host tissues and resident microbiota. These properties vary considerably among yeast strains and therefore represent important selection criteria. Several recently characterized isolates exhibit adhesion-associated characteristics that equal or surpass those of the reference probiotic *S. cerevisiae* var. *boulardii*. For example, *P. kudriavzevii* strain 5S5 displayed strong hydrophobicity together with efficient adhesion to intestinal epithelial cells [36]. Similarly, yeast isolates recovered from traditional fermented horsemeat products, including *C. zeylanoides* C-8, *C. parapsilosis* R-12, and *Rhodotorula alborubescens* R-11, exhibited significantly higher cell surface hydrophobicity, autoaggregation, and coaggregation with pathogenic bacteria than *S. cerevisiae* var. *boulardii* CNCM I-745, suggesting enhanced colonization potential and pathogen exclusion during gastrointestinal transit [33]. Similar adhesion-associated properties have also been described for *C. tropicalis* isolates from raw goat milk, which exhibited autoaggregation indices exceeding 80% together with high tolerance to acidic environments [38].

Another characteristic receiving increasing attention is the ability of probiotic yeasts to inhibit pathogenic microorganisms through multiple complementary mechanisms. *K. marxianus* synthesizes tryptophol acetate, a signaling molecule capable of interfering with bacterial quorum sensing and pathogen-associated behaviors [39]. Mycocins produced by *W. anomalus* inhibit clinically important microorganisms, including *Acinetobacter baumannii*, *Klebsiella pneumoniae*, and *Staphylococcus aureus*, highlighting their broad antimicrobial spectrum [30]. Additional food-derived isolates, including *P. kluyveri* LAR001, *Hanseniaspora uvarum* PIT001, and *C. intermedia* ERQ001, demonstrated antimicrobial activity while simultaneously fulfilling key probiotic selection criteria, including gastrointestinal survival and favorable safety profiles [40]. Similarly, environmental isolates of *L. thermotolerans*, *Metschnikowia reukaufii*, and *Starmerella bombi* inhibited several bacterial and fungal pathogens, indicating that antimicrobial activity is broadly distributed among non-*Saccharomyces* yeasts [37].

Increasing evidence further indicates that probiotic yeasts contribute to host health through antioxidant activity and modulation of cellular redox homeostasis. Several food-associated yeasts produce antioxidant enzymes, carotenoids, glutathione, or other metabolites capable of scavenging reactive oxygen species and limiting oxidative damage [41]. Among recently characterized strains, *D. hansenii* Dh36, *M. caribbica* Mc58, *K. unispora* Kum3-B3, and *M. guilliermondii* Mg112 exhibited high antioxidant capacity together with elevated total phenolic content following fermentation of plant-based beverages, suggesting their suitability for functional food development [9]. Comparable antioxidant activity was observed in yeast isolates recovered from traditional fermented horsemeat products, where all selected strains exhibited radical scavenging activities exceeding 60%, together with cholesterol-lowering capacity [33]. Similar antioxidant properties have previously been described for *D. hansenii* and *K. marxianus*, further supporting the concept that oxidative stress protection represents one of the major functional attributes of probiotic yeasts [42].

Recent investigations also suggest that probiotic yeasts exert metabolic and immunomodulatory effects extending beyond the gastrointestinal lumen. *K. unispora* KU2, originally isolated from Tibetan kefir grains, alleviated patulin-induced intestinal injury in mice by restoring gut microbial composition, reducing inflammatory responses, and increasing intestinal selenium homeostasis through microbiota-dependent upregulation of selenoprotein P (SEPP1). Selenium-enriched *K. unispora* KU2 provided significantly stronger protection than the non-enriched strain, illustrating how probiotic activity may be further enhanced through micronutrient biofortification [43]. These findings demonstrate that probiotic yeasts may influence host physiology not only through direct microbial interactions but also by regulating intestinal microbial communities, inflammatory signaling pathways, micronutrient metabolism, and epithelial homeostasis.

Beyond their direct effects on host physiology, non-*Saccharomyces* yeasts also enhance the nutritional and technological value of fermented foods through diverse metabolic activities. Certain strains produce enzymes and bioactive metabolites that improve nutrient utilization and increase the bioavailability of dietary compounds. For example, a food-derived *S. cerevisiae* isolate recovered from artisanal goat milk exhibited pronounced phytase activity, facilitating phytate hydrolysis and increasing the bioavailability of phosphorus and inositol [44]. Such metabolic activities further expand the functional potential of probiotic yeasts by simultaneously supporting host nutrition and improving the quality of fermented foods.

Although numerous non-*Saccharomyces* yeasts exhibit promising probiotic-associated characteristics, their potential application requires careful safety assessment because probiotic properties are strictly strain-dependent and cannot be inferred solely from species identity. Several taxa investigated as candidate probiotics, including *C. tropicalis*, *C. glabrata*, *C. parapsilosis*, *C. zeylanoides*, and *P. kudriavzevii*, have also been reported as opportunistic pathogens in susceptible individuals. Consequently, the evaluation of novel probiotic yeasts should combine molecular identification with comprehensive phenotypic characterization, including assessment of gastrointestinal survival, antimicrobial susceptibility, hemolytic activity, production of virulence-associated factors, and genomic analyses when appropriate. Recent investigations emphasize that food-derived isolates may display excellent probiotic-associated properties while lacking major virulence determinants, highlighting that safety assessment should be performed at the strain rather than species level [5,33,37].

This concept is well illustrated by recent screening studies of traditional fermented foods. Among sixteen probiotic yeast strains isolated from fermented horsemeat products, all candidates were non-hemolytic and demonstrated favorable biosafety profiles despite belonging to genera that include opportunistic pathogens. Furthermore, selected strains of *C. zeylanoides* exhibited pronounced antioxidant activity, cholesterol-lowering capacity, extracellular antibacterial activity, α-glucosidase inhibition, and angiotensin-converting enzyme (ACE) inhibition, whereas *R. alborubescens* R-11 demonstrated exceptional tolerance to acidic conditions together with strong antioxidant and metabolic activities [33]. These findings emphasize that probiotic functionality and safety are independent characteristics that must both be experimentally validated before a strain can be considered for functional food or therapeutic applications.

**Table 1:** Representative *Saccharomyces* and non-*Saccharomyces* yeasts with reported probiotic-associated traits.

Species (Strain)	Source of Isolation	Representative Probiotic-Associated Traits	References
*Saccharomyces cerevisiae* CL4	Goat milk	Phytase activity; phytate hydrolysis; increased phosphorus and inositol bioavailability	[44]
*Saccharomyces cerevisiae* var. *boulardii* CNCM I-745	Clinical probiotic strain	Clinically validated probiotic; gastrointestinal survival; immunomodulation	[7,22]
*Wickerhamomyces anomalus* HN1	Egyptian cheese	Tolerance to 2% bile salts; gastrointestinal survival	[34]
*Torulaspora delbrueckii* ZIM 2436	Fermented foods	Survival in simulated gastric juice; antioxidant activity	[9,35]
*Pichia kudriavzevii* 5S5	Fermented foods	Gastrointestinal tolerance; adhesion; hydrophobicity	[36]
*Lachancea thermotolerans* Lt101	Plant-based fermented beverage	High viability; functional beverage fermentation	[9]
*Kazachstania unispora* Kum3-B3	Plant-based fermented beverage	Antioxidant capacity; increased total phenolics	[9]
*Kazachstania unispora* KU2	Tibetan kefir grains	Protection against intestinal injury; gut microbiota modulation; SEPP1 upregulation	[43]
*Meyerozyma guilliermondii* Mg112	Plant-based fermented beverage	High viability; antioxidant activity	[9]
*Meyerozyma caribbica* Mc58	Plant-based fermented beverage	Antioxidant activity; high phenolic content	[9]
*Debaryomyces hansenii* Dh36	Plant-based fermented beverage	Antioxidant activity; balanced nutritional profile	[9]
*Candida tropicalis* 12a, 33d	Raw goat milk	High autoaggregation; antioxidant activity	[38]
*Candida zeylanoides* C-8	Fermented horsemeat	Gastrointestinal survival; antioxidant activity; cholesterol lowering; α-glucosidase inhibition; non-hemolytic	[33]
*Rhodotorula alborubescens* R-11	Fermented horsemeat	Exceptional acid tolerance; antioxidant activity; ACE inhibition	[33]
*Starmerella bombi* CMGB-ST1	Plant phyllosphere	Lipolytic activity; antimicrobial activity	[37]
*Metschnikowia reukaufii* CMGB-ST21.2	Plant phyllosphere	Salt tolerance; antimicrobial activity	[37]
*Yarrowia lipolytica* Y6, Y7, Y11	Cold-pressed olive oil	Gastrointestinal survival; hydrophobicity; autoaggregation; coaggregation; antimicrobial, antioxidant and cholesterol-lowering activities; non-hemolytic	[45]

Beyond gastrointestinal health, several non-*Saccharomyces* yeasts possess technological characteristics that further increase their value as functional microorganisms. *D. hansenii* has attracted particular attention because of its Qualified Presumption of Safety (QPS) status, exceptional tolerance to osmotic and environmental stresses, long history of application in fermented foods, and documented antimicrobial, antioxidant, anti-inflammatory, and immunomodulatory properties. Food-derived *D. hansenii* strains have also been shown to stimulate cytokine production by dendritic cells, suggesting their potential contribution to host immune regulation [46,47]. Similarly, *Yarrowia lipolytica* combines metabolic versatility with an established history of food-associated applications. Besides its widespread use in industrial biotechnology, this species naturally participates in cheese ripening, contributes to aroma development through lipolysis and proteolysis, and synthesizes numerous valuable metabolites, including organic acids, carotenoids, polyunsaturated fatty acids, and single-cell proteins. In addition to these technological attributes, increasing experimental evidence indicates that food-derived *Y. lipolytica* strains possess several hallmark probiotic-associated traits, including survival under simulated gastrointestinal conditions, adhesion-related properties, antimicrobial and antioxidant activities, cholesterol-lowering capacity, and favorable safety profiles. These observations further support the growing interest in *Y. lipolytica* as a promising source of both probiotic and postbiotic applications [45].

Overall, these findings demonstrate that probiotic-associated traits are distributed across a broad phylogenetic spectrum of yeasts rather than being restricted to *S. cerevisiae* var. *boulardii*. Contemporary research increasingly focuses on identifying novel food-derived and environmental isolates possessing combinations of desirable functional characteristics, including gastrointestinal survival, antimicrobial activity, adhesion capacity, antioxidant properties, immunomodulation, and favorable safety profiles. Importantly, accumulating evidence consistently demonstrates that these characteristics are strain-specific, emphasizing that probiotic potential cannot be generalized at the species level. Continued integration of comparative genomics, functional phenotyping, metabolomics, and in vivo validation will facilitate the identification of next-generation probiotic yeasts and expand their applications in functional foods, precision nutrition, and microbiome-based therapeutics. At the same time, the diverse repertoire of metabolites, extracellular proteins, cell wall components, and vesicle-associated molecules produced by these yeasts provides a direct conceptual bridge to the yeast-derived postbiotic and parabiotic components discussed in the following sections, further supporting the concept of a yeast-biotic continuum.

## 3. Yeast-Derived Postbiotics: Composition and Functional Diversity

### 3.1. Comparison of Bacterial and Yeast-Derived Postbiotics

According to the International Scientific Association for Probiotics and Prebiotics (ISAPP), postbiotics comprise preparations of intentionally inactivated microorganisms and/or their components that confer a health benefit on the host [2]. Although this definition applies equally to bacterial and yeast-derived preparations, substantial differences in cellular organization result in distinct structural compositions, bioactive components, and mechanisms of action.

Bacterial postbiotics are predominantly composed of fermentation-derived metabolites and cell-associated components, including short-chain fatty acids, organic acids, bacteriocins, exopolysaccharides, peptidoglycan fragments, lipoteichoic acids, and other microbial products that regulate microbial communities, epithelial barrier function, and host immune responses [48,49,50]. Their biological activity is therefore largely mediated by soluble metabolites together with structural fragments released from the bacterial envelope following cellular inactivation.

**Figure 4:**
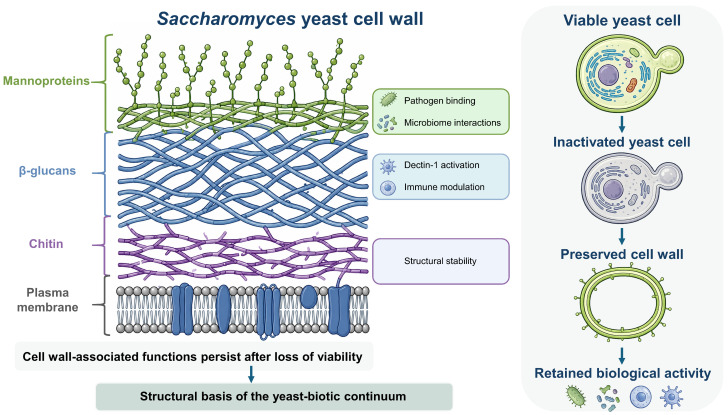
Structural organization of the *Saccharomyces* cell wall and schematic representation of the preservation of cell wall architecture following cellular inactivation. The (**left**) panel illustrates the multilayered organization of the yeast cell wall and the principal biological functions associated with its major structural components. The (**right**) panel depicts the transition from viable to inactivated yeast cells while preserving cell wall architecture and associated biological functions.

In contrast, yeast-derived postbiotics originate from eukaryotic microorganisms with a highly organized cellular architecture. Besides secreted metabolites, proteins, peptides, enzymes, and extracellular vesicles, they retain structurally preserved components of the fungal cell wall that remain largely intact after cellular inactivation (Figure 4). This distinctive organization provides yeast-derived preparations with a combination of structural and molecular bioactive components that is fundamentally different from bacterial postbiotics.

The structural stability of the fungal cell wall represents one of the defining characteristics of yeast-derived postbiotics. Whereas cellular metabolism ceases following microbial inactivation, the multilayered cell wall architecture remains largely preserved, maintaining the spatial organization and biological accessibility of its associated molecules. Consequently, many biological activities associated with yeast-derived preparations are mediated not only by soluble metabolites but also by structurally preserved cell wall components, including mannoproteins, β-glucans and chitin, which remain available for interaction with host tissues after loss of cellular viability.

These structural differences distinguish yeast-derived postbiotics from bacterial preparations and provide the basis for their unique functional diversity. The principal bioactive components of yeast-derived postbiotics, including cell wall polysaccharides, proteins, peptides, extracellular vesicles, and metabolites, are discussed in the following sections.

### 3.2. Key Components of Yeast-Derived Postbiotics

#### 3.2.1. Mannoproteins

Mannoproteins are major glycoprotein constituents of the yeast cell wall and represent one of the principal classes of yeast-derived postbiotic molecules. Localized predominantly within the outer cell wall layer, they contribute to cell wall architecture, surface physicochemical properties, and interactions between yeast cells and their surrounding environment [51,52].

Beyond their structural role, mannoproteins are key mediators of host–microbe and microbe–microbe interactions. Mannan-rich glycans exposed on the yeast cell surface can bind mannose-recognizing adhesins expressed by enteric bacteria, thereby limiting pathogen attachment to intestinal epithelial cells and facilitating pathogen removal from the gastrointestinal tract [53]. Recent structural studies further demonstrated the high-affinity recognition of yeast mannoproteins and mannan by bacterial lectin-like surface proteins, highlighting the molecular specificity underlying these carbohydrate-mediated interactions [54].

Accumulating evidence also indicates that mannoproteins contribute to modulation of the intestinal microbiota. In *S. cerevisiae* var. *boulardii*, metabolic engineering to increase cell wall mannan content significantly enhanced adhesion to *Salmonella enterica* serovar Typhimurium while selectively promoting the growth of the beneficial commensal Bacteroides thetaiotaomicron over *C. difficile*. These findings provide direct experimental evidence that cell wall mannans contribute simultaneously to antimicrobial and prebiotic activities and represent important determinants of probiotic functionality [55].

The biological activity of mannoproteins is largely preserved following cellular inactivation because these glycoproteins remain associated with the structurally intact yeast cell wall. Consequently, mannoproteins continue to participate in interactions with host tissues and microorganisms independently of cellular viability, making them one of the most important functional components of yeast-derived postbiotic preparations.

#### 3.2.2. β-Glucans

Among yeast-derived postbiotic components, β-glucans are the most extensively characterized and biologically validated bioactive polysaccharides. In *S. cerevisiae*, they constitute the predominant structural component of the cell wall and consist primarily of a β-(1→3)-D-glucan backbone with β-(1→6)-linked side branches. This highly branched molecular architecture determines their physicochemical properties, receptor recognition, and biological activity, while structural features such as molecular weight, branching degree, and extraction method substantially influence their functional performance [23,56,57,58].

Unlike many microbial metabolites that disappear after cell death, yeast β-glucans remain structurally stable following thermal or chemical inactivation because they are integral components of the cell wall. Consequently, they preserve their biological activity in postbiotic preparations and represent one of the principal effector molecules responsible for the health-promoting properties of inactivated yeast cells [23,59,60].

The biological functions of yeast β-glucans extend far beyond their structural role. They are recognized by pattern-recognition receptors expressed on innate immune cells, particularly Dectin-1 and complement receptor 3 (CR3), thereby initiating signaling pathways that regulate cytokine production, macrophage activation, dendritic cell maturation, neutrophil function, and phagocytosis. Recent evidence further demonstrates that β-glucans induce immunometabolic reprogramming of innate immune cells and contribute to trained immunity, providing enhanced protection against subsequent microbial challenges while maintaining immune homeostasis [23,61,62,63].

Besides immunomodulation, yeast β-glucans exhibit antioxidant, anti-inflammatory, antimicrobial, prebiotic, and barrier-protective activities. Experimental studies indicate that these polysaccharides reduce oxidative stress through activation of the Dectin-1/Nrf2/HO-1 signaling pathway, attenuate inflammatory responses by modulating NF-κB- and JNK-dependent pathways, promote epithelial barrier integrity, and contribute to gut microbiota homeostasis. Collectively, these mechanisms support their application as multifunctional postbiotic compounds for improving host resilience and intestinal health [23,60,63,64].

Commercially, spent brewer’s yeast generated during beer production represents one of the most abundant, sustainable, and economically attractive sources of β-glucans [65]. The valorization of this brewing by-product aligns with circular bioeconomy principles while providing a scalable raw material for the production of functional food ingredients, nutraceuticals, and biomedical products. Nevertheless, current research emphasizes that extraction procedures and downstream processing significantly affect β-glucan purity, molecular architecture, and consequently their biological efficacy, highlighting the need for standardized production protocols [23,60,66].

Importantly, β-glucans exemplify the yeast-biotic continuum proposed in this review. Although synthesized exclusively by metabolically active yeast cells, their highly ordered polysaccharide network remains intact after cellular inactivation, allowing postbiotic preparations to retain many of the immunomodulatory and host-interactive functions originally associated with viable probiotic cells. Accordingly, β-glucans provide one of the clearest mechanistic links between probiotic and postbiotic states, illustrating how structural components of the yeast cell wall continue to mediate biological effects independently of cell viability.

#### 3.2.3. Bioactive Peptides and Metabolic Products

In addition to structural cell wall components, yeasts produce a diverse repertoire of soluble bioactive molecules during growth, fermentation, and controlled autolysis that may remain biologically active following cellular inactivation [2,67]. These include peptides, proteins, organic acids, polyamines, vitamins, volatile organic compounds (VOCs), and other low-molecular-weight metabolites that contribute to the functional properties of yeast-derived postbiotics [67,68]. These metabolites contribute to microbial interactions, inhibit pathogen growth, regulate cellular redox homeostasis, and influence host physiological responses compounds [68,69].

Among these molecules, bioactive peptides have attracted increasing attention because of their antioxidant, antimicrobial, anti-inflammatory, immunomodulatory, antihypertensive, and hypoglycemic activities. Most yeast-derived bioactive peptides are generated through enzymatic hydrolysis of intracellular proteins during autolysis or controlled processing of yeast biomass, yielding short peptide sequences that retain biological activity after cell inactivation. Peptide-rich yeast hydrolysates have been shown to modulate oxidative stress, inflammatory pathways, and glucose metabolism, highlighting their potential as functional ingredients in food, nutraceutical, and biomedical applications [70,71,72,73]. Recent studies further demonstrate that peptide- and polysaccharide-rich yeast fermentation products can attenuate oxidative stress, suppress pro-inflammatory cytokine production, and modulate cellular signaling pathways associated with inflammation, further supporting their therapeutic potential as postbiotic preparations [74].

Certain extracellular proteins produced by yeasts, particularly mycocins (killer toxins), further expand the functional diversity of yeast-derived postbiotics. These proteins inhibit susceptible fungi through species-specific mechanisms involving disruption of cell wall integrity, membrane permeabilization, or interference with essential cellular processes, illustrating the broad spectrum of biologically active molecules produced by yeasts beyond structural cell wall constituents [75]. Particular classes of metabolites, including short-chain fatty acids, are discussed separately in the following section because of their distinct physiological roles.

From the perspective of the yeast-biotic continuum, peptides and metabolites occupy an intermediate position between living probiotic cells and fully inactivated postbiotic preparations. They originate from active cellular metabolism yet may remain functional independently of cell viability. Consequently, these compounds provide a direct example of how biological activity can persist beyond the living state of the microorganism, reinforcing the concept of functional continuity between probiotic and postbiotic forms.

#### 3.2.4. Short-Chain Fatty Acids (SCFAs)

Short-chain fatty acids (SCFAs) are well-recognized microbial metabolites that play important roles in host metabolism, intestinal homeostasis, and microbiome regulation. Although bacterial fermentation represents the principal source of SCFAs within the gastrointestinal tract, acetate is the predominant SCFA associated with yeast metabolism and is produced by several yeast species, including *S. cerevisiae*, *S. cerevisiae* var. *boulardii*, *Zygosaccharomyces bailii*, and *Brettanomyces* spp. [76,77]. As a stable metabolite that remains biologically active independently of cell viability, acetate represents one of the principal postbiotic molecules produced by yeasts [2,67].

Acetate is generated during carbohydrate fermentation through pyruvate and acetaldehyde metabolism and contributes not only to microbial ecology but also to host physiological regulation [78,79]. Experimental studies have demonstrated that acetate modulates inflammatory responses, improves insulin sensitivity, and regulates lipid and glucose metabolism through activation of G protein-coupled receptors and inhibition of histone deacetylases [80,81]. In addition, acetate activates AMP-activated protein kinase (AMPK), thereby suppressing lipogenic gene expression and promoting cellular energy homeostasis [82].

Beyond its role as a fermentation product, acetate also functions as an important physiological signal in yeasts. Recent work on the probiotic yeast *S. cerevisiae* var. *boulardii* demonstrated that adaptation to elevated acetate concentrations is accompanied by extensive transcriptional and metabolic remodeling affecting membrane organization, cell wall architecture, oxidative stress responses, and central carbon metabolism. These findings highlight acetate as both a major metabolic product and a determinant of stress adaptation in probiotic yeasts, further emphasizing its relevance for postbiotic functionality [77].

Although most evidence regarding the health-promoting effects of SCFAs originates from bacterial systems, increasing evidence indicates that yeast-derived acetate may also contribute to host–microbe interactions through modulation of metabolism, immune responses, and microbial community dynamics. As the predominant SCFA produced by yeasts, acetate therefore provides a mechanistic link between yeast metabolism and the biological activity of yeast-derived postbiotics.

#### 3.2.5. Yeast Extracellular Vesicles (EVs)

Extracellular vesicles (EVs) are membrane-bound nanostructures released by virtually all living organisms, including bacteria, fungi, plants, and animals. They are surrounded by a lipid bilayer and transport a diverse cargo of proteins, lipids, carbohydrates, nucleic acids, metabolites, and signaling molecules that mediate intercellular communication and influence physiological responses in recipient cells [83]. In mammalian systems, EVs are generally classified into exosomes, ectosomes (microvesicles), and apoptotic bodies according to their biogenesis, size, and mechanism of release. Exosomes typically originate from the endosomal pathway through the formation of multivesicular bodies (MVBs), whereas ectosomes are generated by outward budding of the plasma membrane [84,85]. Components of the endosomal sorting complex required for transport (ESCRT), which plays a central role in EV biogenesis, were originally identified and functionally characterized using *S. cerevisiae*, highlighting the fundamental contribution of yeast models to our understanding of vesicle formation [86].

In fungi, EVs perform an additional function because they must traverse the rigid polysaccharide-rich cell wall before reaching the extracellular environment. This process is thought to involve coordinated vesicular trafficking together with localized cell wall remodeling mediated by glucanases, chitin-modifying enzymes, and other hydrolytic proteins, although the precise mechanisms remain incompletely understood [87,88]. Early investigations of fungal EVs therefore focused predominantly on pathogenic species, including *Cryptococcus neoformans*, *C. albicans*, and *Paracoccidioides* spp., where vesicles were shown to transport capsular polysaccharides, virulence-associated proteins, lipids, carbohydrates, and regulatory RNAs involved in host colonization, immune modulation, biofilm formation, and antifungal resistance [89,90,91].

Although these studies established the fundamental biology of fungal EVs, they also created the perception that extracellular vesicles are primarily associated with fungal pathogenicity. However, recent investigations demonstrate that EVs released by probiotic yeasts perform fundamentally different biological functions and represent biologically active components of yeast-derived postbiotics. Because they retain complex mixtures of bioactive molecules while lacking viable cells, probiotic yeast EVs provide a direct mechanistic link between living probiotic microorganisms and cell-free postbiotic preparations.

The first comprehensive characterization of EVs released by the probiotic yeast *S. cerevisiae* var. *boulardii* CNCM I-745 demonstrated that these vesicles are approximately 140 nm in diameter and contain an exceptionally diverse protein cargo comprising more than 1600 identified proteins [92]. Proteomic analysis revealed numerous enzymes involved in carbohydrate metabolism, membrane trafficking, stress responses, and cell wall organization, including glucanases and chitin-associated proteins that may contribute both to fungal cell wall remodeling and to interactions with the host. Importantly, probiotic EVs stimulated IL-1β and IL-8 production in THP-1 macrophage-like cells without inducing detectable toxicity in the *Galleria mellonella* infection model, indicating that these vesicles retain biological activity while maintaining a favorable safety profile [92]. The authors further proposed that EVs derived from probiotic microorganisms should be regarded as postbiotic components capable of providing health benefits without the potential risks associated with the administration of viable microbial cells.

Further evidence supporting this concept was recently provided for the probiotic yeast candidate *P. kudriavzevii*. EVs isolated from this species exhibited a typical fungal size distribution of 100–150 nm and contained 189 proteins involved in metabolism, stress adaptation, vesicular transport, host interactions, and cell wall organization [93]. Functional analyses demonstrated that these vesicles significantly reduced *S. enterica* adhesion and invasion of intestinal epithelial cells, decreased lactate dehydrogenase release, attenuated intracellular and mitochondrial reactive oxygen species production, reduced nitric oxide accumulation, and suppressed the production of pro-inflammatory cytokines, including IL-1β, IL-6, and IL-8, while simultaneously increasing the anti-inflammatory cytokines IL-4 and IL-10. Moreover, EV preparations showed neither cytotoxicity toward Caco-2 intestinal epithelial cells nor detectable toxicity in the *G. mellonella* model, confirming their potential suitability for therapeutic applications [93].

Proteomic analyses of probiotic yeast EVs additionally suggest possible molecular mechanisms underlying these biological effects. Besides glycolytic enzymes frequently detected in fungal vesicles, probiotic yeast EVs contain proteins involved in cell wall remodeling, heat shock proteins, membrane trafficking proteins, elongation factors, and enzymes previously associated with epithelial protection and immune regulation, including alkaline phosphatase, aminopeptidases, invertase, and enzymes participating in polyamine metabolism [92,93]. Many of these proteins have previously been described as moonlighting proteins that perform distinct extracellular functions independent of their canonical intracellular metabolic roles, suggesting that EVs serve not only as transport vehicles but also as functional delivery systems capable of influencing epithelial integrity, microbial competition, and host immune signaling.

Unlike extracellular vesicles produced by pathogenic fungi, which primarily facilitate host invasion and virulence, EVs released by probiotic yeasts appear to function predominantly as carriers of beneficial bioactive molecules involved in immune modulation, epithelial protection, antimicrobial activity, and microbiota communication. Their biological activities closely resemble many of the effects attributed to viable probiotic cells, supporting the hypothesis that EVs mediate an important proportion of probiotic functionality while eliminating the need to administer living microorganisms [92,93]. Consequently, extracellular vesicles represent one of the most promising classes of next-generation yeast-derived postbiotics.

From the perspective of the yeast-biotic continuum proposed in this review, EVs occupy a unique intermediate position between living probiotic cells and conventional postbiotic preparations. They are generated exclusively by metabolically active yeasts yet remain fully functional after separation from the producing cells, preserving complex combinations of proteins, lipids, polysaccharides, metabolites, and signaling molecules within stable membrane structures. This ability to maintain coordinated biological activity independently of cellular viability distinguishes EVs from individual purified postbiotic molecules and positions them as highly attractive candidates for future cell-free microbiome-based therapeutics.

### 3.3. Functional Properties of Yeast-Derived Postbiotics

#### 3.3.1. Immune Modulation

Immune modulation is one of the most extensively documented biological activities of yeast-derived postbiotics. While the immunological effects of probiotic yeasts originate from metabolically active cells (Section 2), increasing evidence indicates that many of these activities are preserved after cellular inactivation through structurally stable cell wall components and secreted bioactive products. Consequently, yeast-derived postbiotics can regulate both innate and adaptive immune responses independently of microbial viability [94].

Among yeast-derived postbiotic components, mannoproteins are the best-characterized immunomodulators. They activate key effectors of innate immunity, including macrophages and natural killer (NK) cells, stimulate lactoferrin secretion, and interact with mannose-binding lectins involved in opsonization and immunophagocytosis. These interactions enhance phagocytic activity, promote complement activation, and contribute to the initiation of non-specific immune responses [95,96]. Mannans further stimulate macrophage proliferation and nitric oxide production, providing an additional mechanism for innate immune activation [97].

Besides mannoproteins, other structural components of the yeast cell wall, particularly β-glucans and chitin, also contribute to immune regulation. Acting through complementary pattern-recognition receptors, these molecules enhance immunocompetence, promote cytokine production, and strengthen host defence, indicating that immunomodulation results from coordinated interactions among multiple cell wall constituents rather than from a single molecule alone [94] (Cerdán-Alduán et al., 2026).

Experimental studies consistently support these mechanisms. Dietary supplementation with *S. cerevisiae* mannoproteins enhanced both specific and non-specific immune responses in Beagle dogs, resulting in increased hydrogen peroxide production by neutrophils following lipopolysaccharide stimulation [97]. Mannoproteins isolated from a mutant *S. cerevisiae* strain (K48L3) activated the Akt-eNOS signalling pathway and stimulated nitric oxide-mediated angiogenesis in human umbilical vein endothelial cells [98]. Mannan significantly increased the expression of the antimicrobial peptide SBD-1 in ovine rumen epithelial cells, demonstrating its capacity to enhance epithelial innate immunity [99].

The immunomodulatory properties of yeast-derived postbiotics have also been demonstrated in animal production systems. Yeast cell wall preparations altered cytokine expression in lipopolysaccharide-challenged broiler chickens [100], whereas supplementation with cell wall preparations derived from *P. guilliermondii* reduced regulatory T-cell (Treg) populations while increasing intestinal interferon-γ (IFN-γ) production during coccidial infection [101]. Similarly, autolysates of *W. anomalus* alleviated intestinal inflammation in Atlantic salmon (*Salmo salar*), whereas preparations from *Cyberlindnera jadinii* and *Blastobotrys adeninivorans* produced considerably weaker responses, highlighting that immunomodulatory efficacy depends on both yeast species and processing method [102].

Recent studies have identified extracellular vesicles (EVs) as an additional class of immunologically active yeast-derived postbiotics. EVs released by the probiotic yeast *S. cerevisiae* var. *boulardii* CNCM I-745 stimulated IL-1β and IL-8 production in THP-1 monocytes without detectable toxicity, indicating their ability to activate innate immune responses while maintaining a favourable safety profile [92]. In parallel, EVs isolated from the probiotic yeast *P. kudriavzevii* attenuated inflammatory responses in intestinal epithelial cells by reducing intracellular reactive oxygen species, nitric oxide, lactate dehydrogenase release, and the production of IL-1β, IL-6, and IL-8, while simultaneously increasing the anti-inflammatory cytokines IL-4 and IL-10 [93]. These findings indicate that EVs not only reproduce several immunomodulatory activities of viable probiotic yeasts but also represent promising next-generation postbiotic preparations capable of mediating host–microbe communication in a cell-free form [50].

Current evidence demonstrates that immune modulation by yeast-derived postbiotics is mediated through complementary mechanisms involving mannoproteins, β-glucans, chitin, and extracellular vesicles. Rather than acting independently, these components regulate innate and adaptive immune responses, reinforcing the concept of the yeast-biotic continuum proposed in this review.

#### 3.3.2. Gut Barrier Support

Yeast-derived postbiotics preserve intestinal barrier integrity through multiple complementary mechanisms, including reinforcement of epithelial tight junctions, maintenance of epithelial architecture, promotion of epithelial repair, and modulation of gut microbiota. These effects have been demonstrated for structurally distinct postbiotic preparations, including heat-killed yeast cells, fermentation-derived postbiotics, cell wall polysaccharides, extracellular vesicles, and soluble proteins [94,103].

Maintenance of epithelial architecture is one of the most consistent effects of yeast-derived postbiotics. Supplementation with *P. kudriavzevii*-derived postbiotics increased jejunal villus height while simultaneously reducing the expression of pro-inflammatory cytokines, indicating coordinated structural protection of the intestinal mucosa [104]. Heat-killed *S. cerevisiae* var. *boulardii* markedly reduced crypt loss, epithelial injury, and inflammatory cell infiltration in dextran sulfate sodium-induced colitis, thereby restoring intestinal barrier morphology [105].

A central mechanism underlying barrier protection is the preservation of epithelial tight junctions. Fermentation-derived *S. cerevisiae* postbiotics significantly upregulated the expression of Claudin-1 and Occludin while reducing pre-weaning diarrhea, demonstrating that reinforcement of epithelial junctions can occur even in the absence of detectable changes in intestinal morphology [106]. Additional in vivo evidence supports the beneficial effects of fermentation-derived yeast postbiotics on gastrointestinal function. Dietary supplementation with *S. cerevisiae* fermentation-derived postbiotic (SCFP) improved nutrient utilization, increased intestinal digestive enzyme activities, enhanced innate immune responses, and significantly increased resistance to *Vibrio parahaemolyticus* infection in Pacific white shrimp, indicating that yeast postbiotics strengthen host intestinal health and disease resilience under aquaculture conditions [107]. Similar effects were reported for heat-killed *S. cerevisiae* var. *boulardii*, which restored the expression of ZO-1 and Occludin in experimental colitis [105]. In addition, mannan-based preparations increased Occludin expression following *E. coli* challenge and improved jejunal barrier integrity, supporting a direct contribution of yeast cell wall carbohydrates to epithelial homeostasis [108].

Barrier-supporting activity has also been demonstrated using functional epithelial models. The commercial postbiotic ABB C22^®^, composed of tyndallized *S. cerevisiae* var. *boulardii*, *S. cerevisiae*, and *K. marxianus*, significantly increased transepithelial electrical resistance (TEER) in Caco-2 monolayers, indicating enhanced epithelial maturation and barrier function. Pretreatment with ABB C22^®^ also reduced rotavirus replication in intestinal epithelial cells, suggesting that improved epithelial integrity was accompanied by increased resistance to enteric viral infection [109]. Similarly, the β-1,3/1,6-glucan-based postbiotic ABB C1^®^ accelerated recovery of epithelial integrity following enterotoxigenic *E. coli* challenge by promoting sustained restoration of TEER [110].

Recent studies further demonstrate that soluble yeast-derived postbiotic molecules directly regulate epithelial barrier homeostasis through defined intracellular signalling pathways. Cell-free supernatant obtained from *S. cerevisiae* var. *boulardii* alleviated experimental colitis, and proteomic analysis identified thioredoxin as a major bioactive component responsible for these protective effects. Purified thioredoxin activated EGFR/Akt signalling, restored the expression of the tight junction proteins ZO-1 and Claudin-3, reduced epithelial apoptosis and oxidative stress, and promoted epithelial repair. Notably, thioredoxin has also been identified in extracellular vesicles released by *S. cerevisiae* var. *boulardii*, suggesting that multiple postbiotic fractions contribute to barrier protection through interconnected molecular mechanisms [111].

Besides direct effects on epithelial cells, yeast-derived postbiotics contribute to barrier homeostasis through modulation of the intestinal microbiota. Heat-killed *S. cerevisiae* var. *boulardii* restored microbial diversity and composition more efficiently than either viable *S. cerevisiae* var. *boulardii* or purified β-glucan in dextran sulfate sodium-induced colitis [105]. *P. kudriavzevii*-derived postbiotics promoted beneficial bacterial genera, including *Lactobacillus*, *Ruminococcus*, *Prevotella*, and *Butyricicoccus*, changes that were associated with improved intestinal health and reduced inflammation [104].

Current evidence indicates that preservation of intestinal barrier integrity by yeast-derived postbiotics results from coordinated actions on epithelial structure, intercellular junctions, intracellular repair pathways, and gut microbial homeostasis. These complementary mechanisms explain how non-viable yeast-derived preparations retain many barrier-protective properties previously attributed to viable probiotic yeasts.

#### 3.3.3. Antimicrobial Effects

Whole yeast-derived postbiotic preparations and individual cell wall components exhibit antimicrobial activity against a broad spectrum of bacterial pathogens. Postbiotics produced from *S. cerevisiae* inhibited the growth of *Listeria monocytogenes*, *Streptococcus mutans*, *Salmonella* Typhi, and *E. coli* both in vitro and in food models, while simultaneously promoting the growth of beneficial probiotic bacteria, demonstrating that antimicrobial activity is retained after microbial inactivation [112].

Mannoproteins are the most extensively investigated antimicrobial components of yeast-derived postbiotics. Mannoproteins isolated from *S. cerevisiae* inhibited the growth of *Escherichia coli* and *Bacillus subtilis* following thermal–alkaline extraction [113]. Preparations obtained from *S. cerevisiae* BY also inhibited *Pseudomonas aeruginosa* and *Staphylococcus aureus* in a concentration-dependent manner [114]. Similar antimicrobial activity was reported for mannoproteins isolated from *S. cerevisiae*, *Metschnikowia reukaufii*, and *Wickerhamomyces anomalus*, which inhibited *P. aeruginosa*, *Proteus mirabilis*, *Salmonella enterica* subsp. *enterica* serovar Enteritidis, *S. aureus*, and *E. coli*, while simultaneously promoting the growth of beneficial bacteria, including [115].

Besides inhibiting bacterial growth, yeast-derived postbiotics interfere with pathogen colonization. Mannoprotein-rich yeast cell wall preparations reduced adhesion of enterotoxigenic *E. coli* to intestinal villi by acting as competitive ligands for bacterial adhesins [116]. Similar preparations also suppressed the growth of *Clostridium perfringens* by extending the lag phase and reducing the maximum bacterial population in a dose-dependent manner [117].

Animal studies support these antimicrobial effects under physiological conditions. Dietary supplementation with yeast cell wall preparations reduced intestinal *Salmonella* colonization in poultry [118], whereas yeast-derived mannan decreased the abundance of *Campylobacter* in pigs while improving intestinal villus morphology [119]. Fermentation-derived *S. cerevisiae* postbiotics also increased resistance of Pacific white shrimp to *Vibrio parahaemolyticus* infection, consistent with enhanced host protection against enteric pathogens [107].

Yeast-derived postbiotics may also enhance the efficacy of conventional antimicrobial therapy. Mannoprotein preparations from *S. cerevisiae* increased the antibacterial activity of tetracycline, gentamicin, ampicillin, and ciprofloxacin against both *P. aeruginosa* and *S. aureus*, supporting their potential use as antibiotic adjuvants [114].

Collectively, available evidence demonstrates that antimicrobial activity of yeast-derived postbiotics results from complementary mechanisms, including direct inhibition of bacterial growth, interference with pathogen adhesion, selective modulation of beneficial microbiota, and potentiation of antibiotic activity. Although mannoproteins remain the best-characterized antimicrobial constituents, whole yeast-derived postbiotic preparations also exhibit broad antimicrobial activity, indicating that multiple bioactive components contribute to this function.

## 4. Bridging the Gap: The Yeast-Biotic Continuum

The evidence discussed throughout Section 2 and Section 3 demonstrates that the biological activity of probiotic yeasts extends beyond cell viability. While probiotics are classically defined as live microorganisms that confer a health benefit on the host [1], many of the biological functions described in this review are retained after cellular inactivation through structurally preserved cell wall components, extracellular vesicles, and secreted bioactive molecules. This expanding evidence has led to the concepts of parabiotics, postbiotics, and microorganism-derived bioactive compounds, emphasizing that microbial viability is only one determinant of biological activity [3,4,120]. Although these concepts have been extensively developed for bacterial systems, their application to yeasts remains comparatively underexplored. Rather than representing separate biological entities, probiotic and postbiotic yeasts can be viewed as successive functional states of the same microorganism. We therefore propose the concept of the yeast-biotic continuum, in which the dominant mechanisms of biological activity gradually shift from metabolism-dependent processes in viable cells to viability-independent activities mediated by extracellular products and structurally preserved cellular components.

At one end of this continuum are metabolically active probiotic yeasts that survive gastrointestinal transit, adapt to environmental stress, and actively interact with both the host and resident microbiota [8,26]. Their biological effects rely primarily on active metabolism, including the production of enzymes, antimicrobial peptides, organic acids, volatile compounds, and other metabolites, as discussed in Section 2 [24,121]. Between viable probiotics and inactivated postbiotics lies an intermediate functional stage represented by extracellular products released from metabolically active cells. As discussed in Section 3, extracellular vesicles and soluble metabolites remain biologically active after their release and therefore extend yeast functionality beyond the producing cell. Extracellular vesicles are particularly illustrative of this transition because they transport complex molecular cargo, including proteins, lipids, polysaccharides, nucleic acids, and metabolites, that mediate host–microbe communication independently of microbial viability [92,93,122]. Moreover, secreted metabolites continue to influence surrounding microbial communities by modulating bacterial virulence, quorum sensing, and interkingdom communication after their release [39,121].

The biological activity of yeast-derived products is further shaped by the surrounding microbial ecosystem. Rather than acting independently, yeast-derived molecules are continuously transformed, consumed, or amplified by resident microorganisms, modifying their bioavailability and physiological effects. Consequently, the final biological outcome reflects dynamic interactions among yeast-derived products, the host, and the intestinal microbiota rather than the activity of individual compounds alone. At the opposite end of the continuum are non-viable yeast cells that preserve structurally stable bioactive molecules despite the loss of metabolic activity. As demonstrated throughout Section 3, these preparations retain immunomodulatory, gut barrier-supporting, and antimicrobial properties through β-glucans, mannoproteins, chitin, extracellular vesicles, and other stable cellular components [4,120]. These findings indicate that many biological activities previously attributed exclusively to viable probiotics are preserved after inactivation and depend primarily on structural integrity rather than microbial viability [114,115,123].

The proposed yeast-biotic continuum provides a unified framework that integrates the mechanisms discussed throughout this review. Instead of viewing probiotics and postbiotics as distinct categories, this model recognizes them as successive functional states in which biological activity is progressively redistributed from living cells to extracellular products and ultimately to structurally preserved cellular components. This perspective better reflects the complexity of yeast–host interactions and may facilitate the rational development of next-generation yeast-based biotherapeutics in which viability represents one, but not the only, determinant of biological function.

### 4.1. Environmental Sensing and Adaptation

During the viability stage, yeast cells function as metabolically responsive biological systems, capable of detecting and adapting to environmental conditions. Exposure to gastrointestinal stressors, including low pH, bile salts, osmotic stress, and oxidative challenges, activates preserved stress response pathways that support cell survival and maintain functional activity [8,26]. Consequently, the biological effects at this stage are due not only to the physical presence of the yeast cells, but also to their ability to respond dynamically to host-related environments. Vital cells can therefore interact directly with epithelial surfaces, compete for ecological niches, and actively participate in metabolic networks related to the microbiota. In addition to the synthesis of individual bioactive compounds, active metabolism constitutes the functional core of the continuum. At this stage, yeast cells continuously convert environmental stimuli into biologically relevant products through interconnected metabolic pathways. Carbon and nitrogen metabolism, redox regulation, amino acid turnover and energy production processes together determine the amount and composition of molecules released into the extracellular environment. Consequently, active metabolism acts as a mechanistic bridge between viable probiotic cells and the subsequent production of cell-independent bioactive factors. Many of the molecules ultimately classified as postbiotic components are derived from metabolic activities occurring prior to cellular inactivation, which highlights the processual rather than product-specific nature of yeast functionality.

### 4.2. Metabolic Activity and Bioactive Compounds Production

Progress along the continuum shifts the dominant mechanism from environmental sensing to a metabolic one. During active metabolism, yeast cells continuously generate and secrete a diverse repertoire of bioactive compounds, including enzymes, peptides, vitamins, antioxidant molecules, and secondary metabolites [121]. Environmental conditions within the gastrointestinal tract further determine how individual stages of the continuum are expressed. Factors such as pH, bile salts, digestive enzymes, oxygen availability, nutrient composition and intestinal transit time influence yeast survival, metabolic activity, release of bioactive factors and accessibility of structural cell wall components. Therefore, the same yeast-derived preparation may exhibit different biological properties depending on the physiological context in which it is delivered. Importantly, the biological significance of this stage lies in the conversion of intracellular metabolic activity into extracellular functions. In this context, the yeast cell acts as a biocatalytic platform capable of modifying the surrounding microenvironment and generating molecules that affect both the physiology of the microorganisms and the host.

### 4.3. Cell-Independent Signaling and Released Factors

Further along the continuum, biological activity becomes progressively separated from the producing cell and increasingly associated with released molecular entities. Secreted proteins, antimicrobial peptides, soluble polysaccharides, metabolites, and extracellular vesicles remain biologically active after release and function independently of cellular viability [4,120]. This stage represents the transition from cell-mediated to molecule-mediated biological activity. Extracellular vesicles exemplify this transition by transporting proteins, lipids, polysaccharides, nucleic acids, and metabolites that mediate host–microbe and microbe–microbe communication [92,93,122]. Released factors therefore provide a mechanistic link between viable probiotic cells and downstream postbiotic preparations.

### 4.4. Inactivated Cells—Structural Recognition

At the final stage of the continuum, metabolic activity ceases, whereas biological activity is maintained through structurally preserved cellular components. Instead of acting through secretion or metabolic adaptation, inactivated yeast cells interact with the host via conserved cell wall structures, including β-glucans, mannoproteins, and chitin-derived polysaccharides. These molecules are recognized by host pattern-recognition receptors and initiate signalling pathways involved in immune regulation, barrier protection, and antimicrobial defence, as discussed in Section 3 [4,26,123]. Accordingly, biological activity at this stage is governed primarily by molecular recognition rather than by cellular metabolism.

## 5. Mechanistic Insights: A Yeast-Specific Perspective

While the yeast-biotic continuum provides a conceptual framework linking living cells, active metabolism, released bioactive factors, and inactivated cellular structures, the biological effects observed by this continuum ultimately result from specific molecular and cellular mechanisms. Importantly, most of the mechanistic models currently used to explain the function of probiotics come from bacterial systems. Although this framework has greatly expanded our understanding of host-microorganism interactions, it does not fully account for the unique biological features of yeasts, including their eukaryotic organization, complex cell wall architecture, distinct secretory pathways, and participation in fungus-bacteria-host interaction networks [8,10,12]. As such, a yeast-specific functional perspective is needed to understand how biological activity is maintained at different stages of the yeast-biotic continuum.

### 5.1. Immunological Recognition of Yeast Cell Wall Components

The yeast cell wall is the main boundary between yeast-derived biotics and the host’s immune system. In contrast to the bacterial cell envelope, yeast walls are mainly composed of β-glucans, mannoproteins, and chitin-derived polysaccharides, which act as potent molecular patterns associated with microorganisms recognized by host immune receptors [51,97,124]. The highly organized structure of the fungal cell wall therefore plays not only a structural role, but is also an important factor determining the immune recognition of the host.

Among these components, β-glucans are considered to be the most thoroughly studied immunologically active molecules of yeast origin. Recognition of β-glucans by pattern recognition receptors, particularly Dectin-1, initiates signaling cascades involving splenic tyrosine kinase (Syk), CARD9-dependent pathways, and NF-κB activation, leading to cytokine production, phagocyte activation, and modulation of innate and adaptive immune responses [123]. Importantly, these interactions occur independently of cellular viability, which explains the immunomodulatory activity often observed in inactivated yeast preparations and yeast-derived postbiotics [4].

In addition to β-glucans, mannoproteins contribute significantly to the biological activity of yeast walls. Mannoproteins are associated with antimicrobial, immunomodulatory, and prebiotic effects and may participate in host recognition processes through interactions with lectin-type receptors and other pattern recognition systems [114,115]. In addition, yeast mannans have been shown to activate signaling pathways involving receptors of the dectin family and distal kinases, indicating that yeast immune diagnosis results from coordinated responses to multiple cell wall structures rather than a single molecule [99].

Taken together, this suggests that immune recognition is one of the main mechanisms linking living cells, inactivated cells, and structural postbiotic preparations in the yeast-biotic continuum. As cell viability decreases, the dominant biological mechanism shifts from active metabolic interactions to receptor-mediated recognition of conserved fungal structures, while biological functionality remains preserved.

### 5.2. Host Epithelial Cell Interactions and Barrier Function

As discussed in Section 3.3.2, yeast-derived biotics preserve epithelial barrier function through complementary mechanisms that include reinforcement of tight junctions, promotion of epithelial repair, modulation of inflammatory signalling, and microbiota-dependent regulation of barrier homeostasis [22,105,106,125]. Within the yeast-biotic continuum, these mechanisms progressively shift from metabolism-dependent interactions of viable cells to signalling mediated by extracellular products and structurally preserved postbiotic components.

### 5.3. Pathogen Interference: Competition, Neutralization and Modulation of Virulence

As discussed in Section 2 and Section 3, pathogen interference is mediated through multiple complementary mechanisms operating at different stages of the yeast-biotic continuum. Viable yeast cells reduce pathogen colonization through competitive exclusion, whereas extracellular proteins, antimicrobial peptides, metabolites, and structurally preserved cell wall components inhibit pathogen growth, neutralize toxins, and modulate quorum sensing and virulence pathways [24,39,121,125]. This progressive shift from cell-mediated competition to molecule-mediated inhibition illustrates how antimicrobial activity is maintained despite the loss of microbial viability.

### 5.4. Fungi, Bacteria and Host Conversations

Traditional probiotic models have mainly focused on direct host-microbe interactions. However, the gastrointestinal tract is increasingly recognized as a complex ecological network involving continuous communication between bacteria, fungi, and host tissues [70].

In this ecosystem, yeasts participate in bidirectional and multidirectional interactions that affect microbial composition, metabolic activity, and host physiology. Studies have shown that probiotic yeast can alter the dynamics of microbial communities and modulate the composition of the gut microbiota, thereby generating indirect effects on host health [126]. On the other hand, resident bacterial populations can modify the availability, transformation and biological activity of yeast-derived metabolites.

Interkingdom interactions may be particularly relevant to understanding the functionality of yeast. Ref. [39] demonstrated that metabolites produced by probiotic yeast disrupt bacterial communication systems and virulence-related signaling pathways, providing direct evidence that yeast can influence bacterial behavior without the need for physical contact. These observations suggest that yeast-derived biotics should be seen not as isolated functional factors, but as participants in complex ecological communication networks involving microorganisms and host tissues at the same time.

## 6. Future Extensions of the Yeast-Biotic Continuum

### 6.1. Engineering Yeast Functionality Across the Continuum

**Figure 5:**
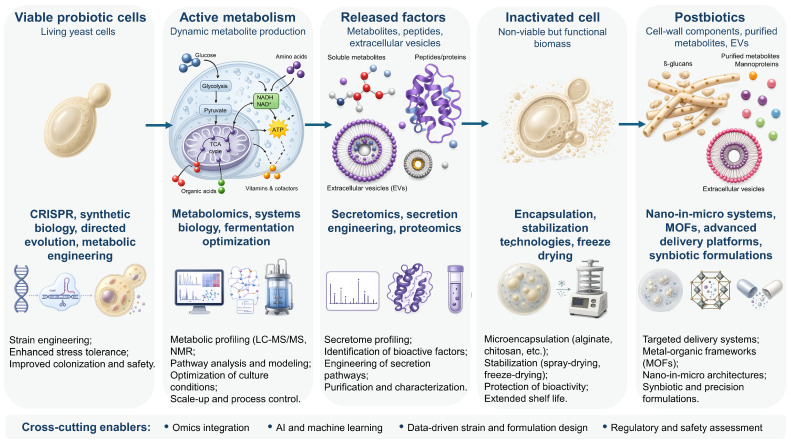
Technological strategies for engineering, preserving and delivering functionality across the yeast-biotic continuum.

The yeast-biotic continuum may be deliberately modified through synthetic biology and genome engineering approaches that alter viability-dependent and viability-independent functions. Such strategies enable the enhancement of specific biological activities associated with different stages of the continuum, including cellular survival, secretion of bioactive molecules, extracellular signaling, and postbiotic functionality (Figure 5).

Recent advances in genetic engineering have transformed probiotic yeasts into programmable platforms capable of delivering tailored therapeutic functions [127]. In *S. cerevisiae* var. *boulardii*, CRISPR-based and conventional genetic engineering approaches have been used to improve antimicrobial, immunomodulatory, and metabolic properties (Table 2). For example, engineered secretion of the antimicrobial peptide leucocin C enhanced antagonistic activity against *L. monocytogenes* [128]. Similarly, deletion of the heme oxygenase gene *HMX1* improved stress tolerance and host-associated fitness in *S. cerevisiae* var. *boulardii* CNCM I-745 [129].

Yeast engineering has also been applied to the development of therapeutic strains targeting metabolic and inflammatory disorders. Expression of pancreatic lipase inhibitor (PLI) peptides in *S. cerevisiae* var. *boulardii* has been proposed as a strategy for anti-obesity interventions [130]. In addition, strains engineered through directed evolution and CRISPR-assisted modification have been developed to enhance responsiveness to extracellular ATP and improve immunomodulatory activity in models of inflammatory bowel disease [131].

Importantly, these approaches demonstrate that individual stages of the yeast-biotic continuum can be rationally engineered. Genetic modifications may influence not only the properties of viable cells, but also the composition of secreted metabolites, peptides, extracellular vesicles, and structural components that remain biologically active after cellular inactivation. Consequently, synthetic biology provides a framework for designing next-generation yeast-derived biotics with functions distributed across multiple stages of the continuum.

### 6.2. Formulation and Delivery of Yeast-Derived Biotics

Beyond their role as probiotics and sources of postbiotic compounds, yeasts have emerged as versatile platforms for the delivery of bioactive molecules. Owing to their eukaryotic cellular organization and unique cell wall architecture, yeast cells can function not only as producers of biologically active compounds but also as carriers capable of protecting, transporting, and releasing functional cargo. This property extends the concept of the yeast-biotic continuum beyond naturally occurring biological activity and into the rational design of yeast-derived functional products.

The growing interest in yeast-based delivery systems is largely driven by the structural characteristics of the yeast cell wall, which is primarily composed of β-glucans, chitin, mannoproteins, and other glycoproteins. These components provide mechanical stability, biocompatibility, and a porous architecture that allows the incorporation of both hydrophilic and hydrophobic compounds [132]. As a result, yeast-derived carriers have been investigated for the delivery of pharmaceuticals, nutraceuticals, dietary supplements, and functional food ingredients, including vitamins, carotenoids, phenolic compounds, flavors, and therapeutic molecules [133].

Within the framework of the yeast-biotic continuum, such technologies are particularly relevant because they enable the preservation and targeted delivery of biologically active entities associated with different stages of yeast functionality, including viable cells, extracellular vesicles, metabolites, peptides, and structural postbiotic components. Consequently, yeast-based delivery systems may serve as a bridge between naturally occurring biological processes and engineered applications designed to enhance the stability, bioavailability, and therapeutic efficacy of yeast-derived biotics.

#### 6.2.1. Encapsulation and Stabilization Strategies

Polymeric hydrogels represent one of the most extensively studied platforms for the encapsulation and delivery of yeast-derived bioactives (Table 2). These three-dimensional polymeric networks are capable of retaining large amounts of water while providing a protective environment for encapsulated cargo [134]. Among the available materials, alginate has received particular attention because of its ability to rapidly form hydrogels through cross-linking with divalent cations such as Ca^2+^. Typically, yeast cells or yeast-derived particles are mixed with alginate solutions and subsequently gelled through extrusion or atomization into calcium-containing media.

Such yeast-in-hydrogel systems have demonstrated enhanced stability and improved release characteristics compared with either hydrogels or yeast carriers alone. In addition to increasing storage stability, alginate-based formulations have been shown to improve gastrointestinal survival of encapsulated microorganisms and facilitate controlled release of biologically active compounds [135]. Their versatility has also been demonstrated in biomedical applications, including oral insulin delivery, where yeast-in-alginate hydrogels enhanced hypoglycemic activity in diabetic animal models [136]. Because conventional alginate hydrogels often exhibit high porosity and limited retention efficiency, secondary coatings such as polydopamine have been applied to improve cargo retention and release control [137].

Another strategy for improving carrier performance involves layer-by-layer (LBL) assembly (Table 2). This approach relies on the sequential deposition of oppositely charged polyelectrolytes onto yeast particles or yeast glucan particles, generating multilayer protective shells with tunable permeability [138]. For example, anthocyanin-loaded glucan particles have been successfully coated using alternating layers of chitosan and negatively charged polymers such as gum arabic, chondroitin sulfate, or dextran sulfate. These multilayer systems enhance the retention of encapsulated compounds, reduce oxygen diffusion, and improve storage stability of sensitive bioactives [139,140,141]. Such approaches illustrate how the natural architecture of yeast-derived particles can be further engineered to generate delivery systems with precisely controlled release characteristics.

#### 6.2.2. Advanced Yeast-Based Delivery Platforms

Although yeast cell walls provide intrinsic protection for encapsulated compounds, their porous structure can permit the diffusion of small molecules, limiting retention efficiency. To overcome this limitation, advanced nano-in-micro systems (Table 2) have been developed in which nanoscale carriers are incorporated into microscale yeast-derived particles. These hybrid platforms combine the high loading capacity and targeting potential of nanocarriers with the structural stability and biocompatibility of yeast glucan particles.

In a typical nano-in-micro design, bioactive compounds are first encapsulated within nanocarriers such as nanoemulsions, nanoliposomes, or solid lipid nanoparticles before being loaded into yeast-derived particles. Such systems improve encapsulation efficiency, enhance retention stability, and provide more controlled release profiles. For example, folate-conjugated, chitosan-coated nanoliposomes containing gefitinib and ZnO quantum dots were incorporated into yeast glucan particles, generating a composite platform with significantly enhanced antitumor efficacy *in vivo* [123]. Cabazitaxel-loaded poly(ε-caprolactone)-lipid hybrid nanoparticles delivered using yeast glucan particles exhibited approximately 5.7-fold higher oral bioavailability than the free drug formulation [142]. Yeast glucan particles have also been used as carriers for other nanoparticle systems, including poly(lactide-co-glycolide) and polyethyleneimine nanoparticles, further demonstrating their versatility as oral delivery platforms [143,144].

More recently, yeast-based delivery systems have been combined with metal–organic frameworks (MOFs) (Table 2), highly porous crystalline materials with tunable physicochemical properties [145]. Through interfacial crystallization processes, MOF layers can be formed directly on yeast surfaces, generating microcapsules capable of sustained, size-selective release of encapsulated compounds [146]. Alternative metal–phenolic coatings based on tannic acid and Fe^3+^ ions have also been developed, providing additional protection against enzymatic degradation, ultraviolet irradiation, and microbial interactions [147]. Despite their considerable promise, MOF-based systems remain limited by susceptibility to acid-induced degradation, which may compromise performance under gastric conditions [57].

Collectively, these advanced delivery platforms demonstrate how the structural properties of yeast can be exploited to create multifunctional carriers capable of transporting and protecting diverse bioactive compounds. Within the yeast-biotic continuum, such technologies expand the practical applications of yeast-derived functionality by enabling the controlled delivery of molecules originating from different functional states of the yeast cell.

### 6.3. Delivery Strategies for Yeast-Derived Biotics

The successful application of yeast-derived biotics depends not only on their biological activity but also on their ability to retain functionality during processing, storage, and gastrointestinal transit. Orally administered probiotic preparations are exposed to numerous environmental stresses, including acidic gastric conditions, bile salts, digestive enzymes, osmotic stress, oxygen exposure, and competition with resident microbiota [13]. These factors can substantially reduce cell viability and compromise the stability of yeast-derived bioactive molecules. Consequently, considerable effort has been devoted to the development of formulation strategies that preserve biological activity and ensure efficient delivery to target sites within the gastrointestinal tract.

Within the framework of the yeast-biotic continuum, these technologies are relevant not only for maintaining the viability of probiotic cells but also for preserving the functionality of extracellular vesicles, metabolites, peptides, and structural postbiotic components. As a result, formulation technologies play a central role in translating yeast-derived biological activity into stable and effective therapeutic, nutritional, and biotechnological products.

#### 6.3.1. Technologies for Improving Stability and Gastrointestinal Survival

Among the available stabilization approaches, immobilization and encapsulation remain the most widely used. Immobilization involves the entrapment of microbial cells within protective matrices that reduce exposure to environmental stresses while maintaining biological activity. Although immobilization has been shown to improve the survival of both bacterial and yeast probiotics, partial exposure of cells at the matrix surface may limit the overall protective effect [148,149].

Encapsulation provides a more comprehensive protective strategy by enclosing viable cells within polymeric, protein-based, or lipid matrices that shield microorganisms from adverse environmental conditions while enabling controlled release at the target site [11,150]. Microencapsulation is particularly suitable for probiotic applications because microbial cells are typically within the micrometer size range. Various encapsulating materials, including alginate, chitosan, starch, κ-carrageenan, gelatin, milk proteins, and cellulose derivatives, have been employed to improve stability and gastrointestinal delivery [151,152,153].

Several encapsulation strategies are available for yeast-based formulations. Among them, extrusion is one of the simplest and most widely used techniques because it operates under mild conditions and generally preserves high cell viability. Alginate-chitosan microspheres produced by extrusion protect *S. cerevisiae* var. *boulardii* under highly acidic conditions while enabling pH-dependent release in intestinal environments [154,155]. Emulsion-based encapsulation further improves the storage stability and gastrointestinal survival of probiotic yeasts [156].

Drying technologies represent another important group of stabilization methods. Spray drying offers rapid and cost-effective large-scale production but may negatively affect viability because of heat and dehydration stress. Optimization of process parameters, particularly inlet temperature, can substantially improve survival rates [157,158]. In contrast, freeze drying (Table 2) generally provides higher survival because it operates under milder thermal conditions, although cryoprotectants such as trehalose, lactose, and polysaccharides are often required to minimize freezing-associated damage [138,159]. Additional approaches, including spray chilling, fluidized-bed drying, and supercritical-fluid technologies, have further expanded the toolbox available for preserving yeast functionality during storage and delivery.

#### 6.3.2. Advanced Probiotic Formulations

Recent developments increasingly focus on multifunctional formulations that combine stabilization with enhanced biological performance. One prominent example is the co-encapsulation of probiotics with prebiotics such as inulin, fructooligosaccharides, and lactulose. These formulations not only protect microbial cells during gastrointestinal transit but also provide metabolic substrates that support microbial activity after delivery. Such systems are commonly referred to as synbiotics and represent a promising strategy for improving both survival and functional efficacy [160,161].

Advances in formulation science are also enabling the development of delivery systems specifically tailored to different stages of the yeast-biotic continuum. While some approaches are designed to maximize the survival of viable probiotic cells, others aim to preserve secreted metabolites, extracellular vesicles, peptides, or structural postbiotic components. Consequently, modern formulation technologies increasingly support the concept that biological functionality can be maintained and delivered independently of cellular viability.

Future developments are expected to integrate encapsulation technologies with synthetic biology, advanced biomaterials, and precision microbiome interventions (Table 2). Such approaches may facilitate the design of yeast-derived biotics with improved stability, targeted release characteristics, and enhanced therapeutic performance, thereby expanding the practical applications of the yeast-biotic continuum.

### 6.4. Omics-Guided Development of Yeast Biotics

#### 6.4.1. Metabolomics

Metabolomics provides a powerful framework for characterizing functional states across the yeast-biotic continuum (Table 2). Because many biological effects associated with probiotic yeasts are mediated by secreted metabolites rather than by the mere presence of viable cells, comprehensive metabolic profiling can help identify the molecules responsible for host modulation, microbial interactions, and postbiotic activity.

Recent untargeted LC–MS/MS analyses demonstrated that both *S. cerevisiae* var. *boulardii* and *S. cerevisiae* substantially altered the metabolism of *S. mutans* and *C. albicans* in planktonic co-culture systems. Both yeasts induced widespread metabolic reprogramming, including the downregulation of carbohydrate and amino acid metabolism and the modulation of purine biosynthesis pathways. These changes were associated with reduced acidogenic potential and altered microbial interactions, illustrating how yeast-derived metabolites can influence surrounding microbial communities [162].

Metabolomics has also been applied to investigate the behavior of probiotic yeasts in functional food formulations. In ice cream containing *S. cerevisiae* var. *boulardii* CNCM I-745, metabolomic profiling revealed progressive changes during frozen storage, with numerous differentially expressed metabolites associated with organic acid metabolism, amino acid metabolism, and carbohydrate utilization [163]. Similarly, metabolomic analysis of synbiotic yoghurt supplemented with inulin identified metabolites including hydroxyisocaproic acid, indolelactic acid, trehalose, raffinose, maltohexaose, and D-glucuronic acid, which may contribute to both probiotic persistence and product functionality [50].

Within the context of the yeast-biotic continuum, metabolomics provides a direct means of characterizing the transition from active metabolism to metabolite-mediated biological activity and therefore represents a key tool for identifying functional postbiotic components and biomarkers of efficacy.

#### 6.4.2. Secretome Analysis

Secretome analysis complements metabolomics by characterizing the extracellular proteins, peptides, enzymes, and signaling molecules released by yeast cells (Table 2). Because many yeast-associated biological activities are mediated through secreted factors, the secretome represents a critical interface between viable probiotic cells and viability-independent functional products.

Recent work using engineered *S. cerevisiae* var. *boulardii* demonstrated the potential of secretome-focused approaches for the development of next-generation yeast biotics. Using a *C. difficile* toxin A-neutralizing peptide as a model therapeutic, researchers combined expression cassette optimization, secretion signal engineering, and genome modification to substantially enhance extracellular peptide production. Proteomics-guided identification of secretion bottlenecks further enabled the construction of a quadruple protease-deficient strain capable of producing more than 5 g L^−1^ of the therapeutic peptide, representing over a tenfold improvement compared with the parental strain [164].

These studies illustrate how secretome analysis can support the rational engineering of yeast-derived biological functions. Beyond therapeutic proteins, similar approaches may facilitate the identification and optimization of naturally secreted enzymes, antimicrobial peptides, immunomodulatory proteins, and extracellular vesicle-associated cargo. Consequently, secretomics provides an important analytical bridge between probiotic activity and the generation of functional postbiotic products within the yeast-biotic continuum.

**Table 2:** Representative technologies supporting different stages of the yeast-biotic continuum.

Technology	Continuum Stage(s) Targeted	Main Purpose	Representative Example	References
Synthetic biology and CRISPR engineering	Viable cells	Introduction of novel therapeutic functions	CRISPR-engineered *S. cerevisiae* var. *boulardii*; HMX1 deletion; PLI expression; eATP-responsive strains	[127,129,130,131]
Polymeric hydrogels	Viable cells	Protection during storage and gastrointestinal transit	Alginate hydrogels for probiotic yeasts	[135,136]
Layer-by-layer microcapsules	Viable cells, postbiotic cargo	Improved retention and controlled release	Chitosan/dextran sulfate multilayer coatings	[138,141]
Nano-in-micro systems	Metabolites, therapeutic cargo	Enhanced bioavailability and targeted delivery	Nanoliposome-in-yeast systems; cabazitaxel-loaded glucan particles	[142,165]
Metal–organic framework (MOF) coatings	Viable cells and bioactive cargo	Controlled release and environmental protection	ZIF-8/yeast and CuBTC/yeast microcapsules	[146]
Encapsulation technologies (extrusion, emulsions, spray processes)	Viable cells	Increased survival during processing and gastrointestinal transit	Chitosan-coated alginate microspheres containing *S. cerevisiae* var. *boulardii*	[154,157]
Freeze drying	Viable cells, postbiotic preparations	Long-term stabilization	Freeze-dried multilayer-coated *S. cerevisiae* var. *boulardii*	[166]
Synbiotic formulations	Viable cells and metabolites	Simultaneous protection and metabolic support	Inulin-containing yeast formulations	[160]
Metabolomics	Active metabolism, released factors	Identification of functional metabolites and metabolic interactions	LC-MS/MS profiling of *S. cerevisiae* var. *boulardii* and *S. cerevisiae*	[50,162,163]
Secretome analysis	Released factors	Characterization and optimization of secreted bioactives	NPA-secreting engineered *S. cerevisiae* var. *boulardii*	[164]
Precision yeast biotics	Entire continuum	Personalized and function-guided interventions	Integration of engineering, formulation and omics approaches	Conceptual framework

### 6.5. Toward Precision Yeast Biotics

The emergence of precision microbiome interventions is creating new opportunities for the development of yeast-derived biotics tailored to specific clinical, nutritional, and microbiological contexts. Several intrinsic characteristics make probiotic yeasts particularly attractive candidates for such approaches. Unlike many bacterial probiotics, probiotic yeasts exhibit high tolerance to gastric acidity and bile stress, enabling efficient gastrointestinal delivery. In addition, they remain unaffected by antibacterial antibiotics and therefore retain functionality during antibiotic treatment, making them valuable for the prevention and management of antibiotic-associated dysbiosis and diarrhea.

Importantly, the yeast-biotic continuum provides a conceptual framework for precision biotics that extends beyond the administration of viable cells alone. Depending on the intended application, therapeutic functionality may be derived from living probiotic cells, secreted metabolites, extracellular vesicles, purified cell-wall components, or defined postbiotic preparations. Advances in synthetic biology, omics technologies, and formulation science increasingly enable the selective optimization of these individual functional layers.

Future precision yeast biotics may therefore combine strain-specific engineering, targeted delivery systems, and molecular characterization of bioactive components to generate interventions tailored to particular host populations, disease states, or microbiome configurations. In this context, the yeast-biotic continuum offers a framework for integrating viable and non-viable functional products into a unified strategy for microbiome-based health interventions.

## 7. Challenges, Limitations and Conclusions

Despite the therapeutic potential of probiotic yeasts, their clinical and industrial implementation faces significant biological, analytical, and regulatory issues. A primary regulatory challenge is the lack of standardized terminology, particularly for postbiotics, where the absence of a global consensus on inanimate microbial fractions and yeast-derived metabolites delays product approval and commercialization [3]. Analytically, although the yeast secretome is remarkably rich, the isolation, purification, and characterization of individual bioactive macromolecules responsible for specific health benefits remain highly demanding [12]. A major clinical gap is the deficit of human trial data for species other than *S. cerevisiae* var. *boulardii*. While numerous non-*Saccharomyces* strains show promising *in vitro* functional benefits, they frequently fail to reproduce these results in human clinical models [46]. Consequently, only *S. cerevisiae* var. *boulardii* is definitively established as a clinical probiotic [125], whereas promising candidates like *K. lactis* and *D. hansenii* require extensive randomized, double-blind clinical trials to validate their benefits [167]. Patient biosecurity remains a critical concern. Although yeasts are safe for immunocompetent populations, rare instances of opportunistic systemic fungemia occur in high-risk demographics, including patients undergoing enteral nutrition [168], those with central venous catheters, or critically immunocompromised states [169]. This necessitates strict clinical monitoring and mandatory antifungal susceptibility screening for all candidate strains [125]. Importantly, probiotic functionality is a strain-specific phenomenon rather than a species-conserved trait [1]. Minor genetic variations within the accessory genome alter the phenotypic expression of surface adhesins, secretory pathways, and immunomodulatory profiles [126]. Furthermore, in species like *T. delbrueckii*, essential probiotic attributes- such as gastroprotective tolerance and antioxidant potential- are restricted to specific, rigorously selected isolates rather than the entire species [34]. Therefore, testing just the species is not enough. Full genomic sequencing and clinical validation of each specific strain are necessary to ensure safety and effectiveness [1,170].

Despite these challenges, the available evidence supports the concept that the biological functions of yeasts are distributed across multiple functional states rather than being restricted to metabolically active cells. The yeast-biotic continuum proposed in this review integrates viable probiotic cells, extracellular vesicles, secreted metabolites, and structurally preserved postbiotic components into a unified framework describing the progressive redistribution of biological activity during the transition from viability to cellular inactivation. This framework provides a mechanistic basis for interpreting the complementary functions of yeast-derived biotics and may facilitate the harmonization of currently fragmented terminology. Further progress will depend on standardized methodologies for postbiotic characterization, mechanistic validation of individual bioactive components, and well-designed clinical studies evaluating both established and emerging probiotic yeast species.

## Data Availability

No new data were created or analyzed in this study. Data sharing is not applicable to this article.
